# Competitive Ability and Fitness Differences between Two Introduced Populations of the Invasive Whitefly *Bemisia tabaci* Q in China

**DOI:** 10.1371/journal.pone.0100423

**Published:** 2014-06-19

**Authors:** Yi-Wei Fang, Ling-Yun Liu, Hua-Li Zhang, De-Feng Jiang, Dong Chu

**Affiliations:** Key Lab of Integrated Crop Pest Management of Shandong Province, College of Agronomy and Plant Protection, Qingdao Agricultural University, Qingdao, China; Volcani Center, Israel

## Abstract

**Background:**

Our long-term field survey revealed that the *Cardinium* infection rate in *Bemisia tabaci* Q (also known as biotype Q) population was low in Shandong, China over the past few years. We hypothesize that (1) the *Cardinium*-infected (*C* ^+^) *B*. *tabaci* Q population cannot efficiently compete with the *Cardinium*-uninfected (*C* ^−^) *B*. *tabaci* Q population; (2) no reproductive isolation may have occurred between *C* ^+^ and *C* ^−^; and (3) the *C* ^−^ population has higher fitness than the *C* ^+^ population.

**Methodology and Results:**

To reveal the differences in competitive ability and fitness between the two introduced populations (*C* ^+^ and *C* ^−^), competition between *C* ^+^ and *C* ^−^ was examined over several generations. Subsequently, the reproductive isolation between *C* ^+^ and *C* ^−^ was studied by crossing *C* ^+^ with *C* ^−^ individuals, and the fitnesses of *C* ^+^ and *C* ^−^ populations were compared using a two-sex life table method. Our results demonstrate that the competitive ability of the *C* ^+^ whiteflies was weaker than that of *C* ^−^. There is that no reproductive isolation occurred between the two populations and the *C* ^−^ population had higher fitness than the *C* ^+^ population.

**Conclusion:**

The competitive ability and fitness differences of two populations may explain why *C* ^−^ whitefly populations have been dominant during the past few years in Shandong, China. However, the potential role *Cardinium* plays in whitefly should be further explored.

## Introduction

The sweet potato whitefly, *Bemisia tabaci* (Gennadius) (Hemiptera: Aleyrodoidea), is a major crop pest [1], [2] and this species complex contains at least 31 cryptic species identified based on mitochondrial cytochrome oxidase I (mtCOI) sequences and crossing experiments [3], [4]. The best known species are MEAM1 (commonly known as biotype B, hereafter referred to as *B*. *tabaci* B or B) and MED (commonly known as biotype Q, hereafter referred to as *B*. *tabaci* Q or Q) because both are extremely invasive and globally distributed [3]. *B. tabaci* Q was first detected on the ornamental poinsettia (*Euphorbia pulcherrima* Willd.) in China in 2003 [5]. Since then, it has gradually displaced *B*. *tabaci* B which was introduced in the mid-1990s. Since 2008, *B. tabaci* Q has become the dominant whitefly species in most regions of China [6]–[9].

The symbiont *Cardinium* was first found in cell cultures established from the tick *Ixodes scapularis* Say [10] and was named *Candidatus* Cardinium hertigii by Zchori-Fein *et al*. [11]. *Cardinium* can alter the reproduction of its hosts by feminization [12], [13], parthenogenesis [14], or cytoplasmic incompatibility of infected hosts [15]–[17]. Prior studies showed that infection with this symbiont can improve the fitness of its host [18]–[20]. Harris *et al*. [21] reported that *Cardinium* infection frequency can increase greatly within the wasp *Encarsia pergandiella* population after nine generations. However, our long-term field survey revealed that the *Cardinium* infection rate in *B. tabaci* Q populations was low (7.6% to17.3%) in Shandong, China, during 2006–2009 [22], though the whitefly may have 10–12 generations per year in this area. Pan *et al*. [23] also reported that the infection frequency of *Cardinium* in *B. tabaci* Q was not very high (16%) in 61 localities in 19 provinces of China in 2009.

On the basis of these data, we hypothesize that (1) the *Cardinium*-infected (*C* ^+^) *B*. *tabaci* Q population cannot efficiently compete with the *Cardinium*-uninfected (*C* ^−^) *B*. *tabaci* Q population; (2) no reproductive isolation may have occurred between *C* ^+^ and *C* ^−^, and if there had been reproductive isolation between them, *C* ^+^ would have been completely displaced by *C* ^−^ after long-term coexistence in the field; and (3) the *C* ^+^ population has higher fitness than the *C* ^−^ population.

To reveal the differences in competitive ability and fitness between the two introduced populations (*C* ^+^ and *C* ^−^), competition between *C* ^+^ and *C* ^−^ was examined over several generations. Subsequently, the reproductive isolation between *C* ^+^ and *C* ^−^ was studied by crossing *C* ^+^ with *C* ^−^ individuals, and the fitnesses of *C* ^+^ and *C* ^−^ populations were compared using a two-sex life table method [24].

## Materials and Methods

### Ethics Statement

The research complies with all laws of the country (China) in which it was performed and was approved by the Department of Science and Technology of the Qingdao Agricultural University, China (permit number: 20110712).

### *Bemisia Tabaci* Laboratory Population

The stock population of *B*. *tabaci* was obtained from laboratory colonies established from prior field collections. The *C* ^+^ and *C* ^−^ populations were provided cotton plants and maintained in isolated whitefly-proof screen cages in a greenhouse under controlled lighting and constant temperature (27±1°C) for about ten generations. The primary symbiont, *Porteria*, as well as secondary symbionts belonging to the genera *Arsenophonus*, *Cardinium*, *Hamiltonella*, *Rickttisia*, and *Wolbachia*, have been detected in *B*. *tabaci* Q [22], [25]–[27]. Using the specific primers of the primary symbiont, *Portiera,* and the secondary symbionts, *Arsenophonus, Cardinium, Fritschea, Hamiltonella, Rickettsia,* and *Wolbachia*, we found that the *C* ^+^ and *C* ^−^ populations were also infected with *Portiera* and *Hamiltonella.* Both *C* ^+^ and *C* ^−^ populations were maintained in separate cultures on potted cotton plants, Lu-Mian-Yan 21 cultivar. The purity of each of the cultures was monitored every 30 days by sampling 20 adults using PCR. Cotton plants were cultivated in 1.5 L plastic pots with nutritional soil and enclosed in whitefly-proof screen cages under controlled light and temperature in a screen house. Three pesticide-free, insect-free, young potted cotton plants were used in the large cages (40 cm×25 cm×50 cm). Plants were at the five to seven fully expanded true leaf stage. Plants were watered and replaced as necessary. All experiments were conducted in controlled climate chambers (27±1°C, 16L: 8D, and 60±5% RH).

### *Bemisia Tabaci* Species Determination and Detection of *Cardinium*

Adult whiteflies were collected with a hand-held aspirator, preserved immediately in 95% ethanol, and stored at −20°C until processing. Genomic DNA was extracted from individual adult whiteflies of each collection using the DNAzol kit (Molecular Research Center, Inc., Cincinnati, OH) and stored at −20°C for subsequent use. The cleavage amplified polymorphic sequence (CAPS) of the mtCOI gene was used to determine the species of *B*. *tabaci*. The primers used for detection of the species were C1-J-2195 (5′-TTGATTTTTTGGTCATCCAGAAGT-3′) [28] and R-BQ-2819 (5′-CTGAATATCGRCGAGGCATTCC −3′) [29]. All PCR reactions were performed in 20 µl buffer containing 2 µl 10× buffer, 1.5 mM MgCl_2_, 0.2 µM of each primer, 0.2 µM dNTPs, 1 unit Taq DNA polymerase, and 2 µl template DNA. Reaction conditions were as follows: 1 cycle of 94°C for 5 min, 35 cycles of 94°C for 1 min, 52°C for 1 min, 72°C for 1 min, and final extension at 72°C for 10 min. The presence of mtCOI amplicons was visualized by electrophoresis in 1.0% agarose gel electrophoresis and ethidium bromide staining. The mtCOI fragment (623 bp) was cleaved using the restriction endonuclease *Vsp*I [30]. Aliquots of the PCR products (13 µl) were each digested with 5 U *Vsp*I (in 20 µl total reaction volume) at 37°C for 2 h. Specimens whose mtCOI fragments were cut by *Vsp*I were identified as *B*. *tabaci* Q.

PCR detection of *Cardinium* was performed using the primers Car-sp-F (5′-CGG CTT ATT AAG TCA GTT GTG AAA TCC TAG-3′) and Car-sp-R (5′-TCC TTC CTC CCG CTT ACA CG-3′). All PCR reactions were performed in 20 µl of reaction buffer containing 1×buffer, 0.16 mM of each dNTP, 0.5 mM of each primer, 0.5 unit *Taq* DNA polymerase, and 2 µl template DNA. Reaction conditions were as follows: 1 cycle of 95°C for 1 min, 35 cycles of 95°C for 30s, 57°C for 30 s, 72°C for 1 min, and final extension at 72°C for 5 min. All PCR reactions included a negative control (sterile water instead of DNA) to detect DNA contamination, and a positive control (DNA from previous sequencing) to prevent false negatives. The PCR products (544 bp) were electrophoresed in a 1.0% agarose gel in TAE [31].

### Identification and Analysis of Orthologous Genes between *C^+^* and *C* ^−^ Populations

To reveal genetic divergence between *C^+^* and *C^−^* populations, orthologs of *cytochrome P450* genes in the transcriptomes of these populations were analyzed. About 20 ug of total RNA (≥300 ng/ul) from each population (*C^+^* or *C^−^*) was sent to the Shanghai Sangon Institute for library preparation and sequencing on an Illumina HiSeq2000. Raw reads was obtained and *de novo* transcriptome assembly was done with the short-read assembly program trinity [32]. Pairs of sequences longer than 1000 bp that mapped unambiguously to *cytochrome P450* in the Swissprot database (E value<1e^−5^) were selected as *cytochrome P450* genes. Some *P450* genes such as *P450 4C1* gene has high variation in *B. tabaci* cryptic species [33].

### Competition between *C* ^+^ and *C* ^−^ Populations

To compare the competitive abilities of *C* ^+^ and *C* ^−^ populations, we conducted a cage experiment and followed the frequencies of *Cardinium* infection in whiteflies raised on cotton over ten generations. To observe changes in the relative proportion of *C* ^+^ and *C* ^−^ individuals, 30 pairs of *C* ^+^ and 30 pairs of *C* ^−^ newly emerged adults whitefly cultures were released into a cage and the infection frequency of *Cardinium* was monitored every 25 days (approximately one generation) by sampling 50 adults using PCR. Infection frequency monitoring of *Cardinium* started from the 75^th^ day. Three replicates were carried out for this study.

### Crossing Experiments

To examine the possibility of reproductive isolation between *C* ^+^ and *C* ^−^, we carried out crossing experiments between *Cardinium*-infected and uninfected populations, ♀*C* ^−^×♂*C* ^−^, ♀*C* ^−^×♂*C* ^+^, ♀*C* ^+^×♂*C* ^−^, and ♀*C* ^+^×♂*C* ^+^, using virgin whiteflies. To obtain newly emerged unmated adults for experiments, adults were allowed to emerge in isolation and were kept individually before crossing. In the evening, cotton leaves with whitefly pupae (late 4^th^ instar nymphs with red eyes) were cut from plants, and individual pupae with the attached portion of the leaf were placed into a Petri dish. To maintain humidity, a moist filter paper was put on the bottom of the Petri dish. The next morning (at 7∶00 am), the newly emerged adults were collected and sexed using a stereomicroscope [34].

For each cross, newly emerged females were individually transferred onto a cotton seedling as described by Li *et al.* [35] together with three adult males. Each female was allowed to lay eggs for 72 h. Adults were then collected using a small aspirator and stored at −20°C for later PCR confirmation of identity. To determine the sex ratio, the eggs laid by each female were observed daily until adult emergence and the newly emerged adults were collected and sexed using a stereomicroscope. Data were analyzed using a one-way analysis of variance (ANOVA) and means were compared using a least significant difference (LSD) test. To normalize the data, an arcsine transformation was used for sex ratio.

### Fitness Assessment of *C* ^+^ and *C* ^−^ Population using the Two-Sex Life Table Method

To reveal differences in the fitnesses of *C* ^+^ and *C* ^−^, we analyzed the demographic parameters of the population using the two-sex life table method. For the life table study, the rearing containers were made of plastic pots (11.5 cm top diameter, 7.8 cm bottom diameter, and 15.5 cm height), with inverted plastic cups (11.5 cm top diameter, 7.8 cm bottom diameter, and 15.5 cm height) used as covers. The bottom of the plastic cup was cut out and covered with fine mesh cloth for ventilation. A cotton seedling at the two-true-leaves stage was used in this study, only one true leaf was kept on the seedling and the other leaf was removed.

For the life table study, approximately 15 pairs of whiteflies were transferred into rearing containers with a cotton seedling. Eggs laid within 24 h were collected and 15 eggs were used for the life table study. Other eggs were removed. For the *C* ^+^ or *C* ^−^ populations, 15 replicates were carried out. The developmental stage and survival status of individual eggs were recorded daily starting at day 14, which corresponded to the day before adult emergence. Using this method, the survival rate of eggs was determined.

To determine fecundity, newly emerged whiteflies were collected and paired in egg-laying units [35]. The whiteflies were checked daily for survival and fecundity until the death of all individuals. Cotton seedlings were replaced daily.

For each population, the life history raw data of all individuals were analyzed based on the age-stage, two-sex life table theory [24] and the method described by Chi [36]. The software TWOSEX-MSChart [36] (available at http://140.120.197.173/Ecology/download/Twosex-MSChart.rar) was used for raw data analysis. The age-stage specific survival rate (*s_xj_* where *x*  =  age and *j*  =  stage), age-stage specific fecundity *(f_xj_*), age-specific survival rate (*l_x_*), age-special fecundity (*m_x_*), age-stage life expectancy (*e_xj_*), reproductive value (*v_xj_*), preoviposition period of the adult female (APOP), and total preoviposition period of the female from birth (TPOP) were calculated. Among these parameters, *s_xj_* represents the probability that a newborn will survive to age *x* and stage *j*, and *f_xj_* represents the mean number of offspring produced by a female of age *x*. In the age-stage, two-sex life table, according to Chi and Liu [37], *l_x_* is estimated as 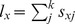, and *m*_x_ is estimated as
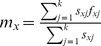
, where *k* is the number of stages. The age-stage life expectancy (*e_xj_*) is the length of time that an individual of age *x* and stage *j* is expected to live. The life expectancy for an individual of age *x* and stage *y* was calculated as 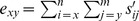, where *n* is the number of age groups, *m* is the number of stages, and 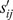
is the probability that an individual of age *x* and stage *y* will survive to age *i* and stage *j* and is calculated by assuming 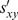 = 1 [37].

Population parameters, including the intrinsic rate of increase (*r*), finite rate of increase (*λ*), net reproductive rate (*R*_0_), and mean generation time (*T*), were calculated as well. A bootstrap method [38] was used to estimate the standard errors of the population parameters. Among these parameters, the intrinsic rate of increase was estimated using the iterative bisection method from the Euler-Lotka equation (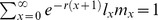) with age indexed from 0 [39]. The net reproduction rate (*R_0_*) is calculated as 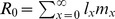. The mean generation time is defined as the length of time that a population needs to increase *R_0_* fold of its size (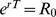 or 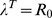) at a stable age distribution, and the mean generation time was calculated as 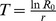. Student’s *t-test* was used to determine differences in developmental times, fecundity, and population parameters between the *C* ^+^ and *C* ^−^ populations.

## Results

### Identification and Analysis of Orthologous Genes between *C^+^* and *C* ^−^ Populations

In the preliminary analysis, 40 *cytochrome P450* genes were identified (Table S1). Based on the *cytochrome P450* genes identified in this study, the average identity between *C^+^* and *C^−^* populations was 99.7% (range, 98.4% to 100.0%) (Table S1), indicating that the genetic backgrounds of these two populations are highly similar.

### Competition between *C* ^+^ and *C* ^−^ Populations

**Figure 1:**
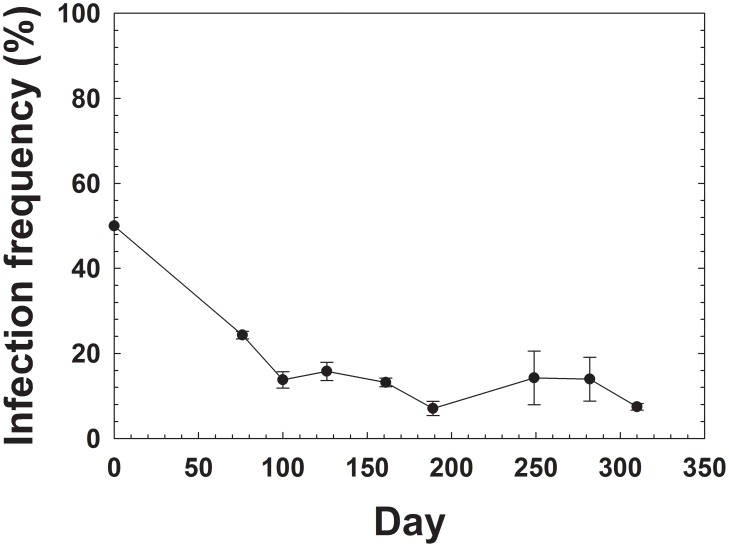
Percentages of *Cardinium*-infected *B. tabaci* Q (*C^+^*) in a mixed cohort.

The changes in the relative ratio of *C* ^+^ and *C* ^−^ individuals within 310 days (approximately 11 generations) are shown in Fig. 1. The mixed cohort began with 50% *C* ^+^ and 50% *C* ^−^ individuals. The relative ratio of *C* ^−^ individuals reached 76% after 75 days and increased steadily over time, reaching 93% after 310 days. By contrast, the relative ratio of *C* ^+^ individuals decreased from the initial 50% to 24% after 75 days and remained between 7% and 20% thereafter.

### Sex Ratio among Crosses between *C* ^+^ and *C* ^−^ Populations

**Figure 2:**
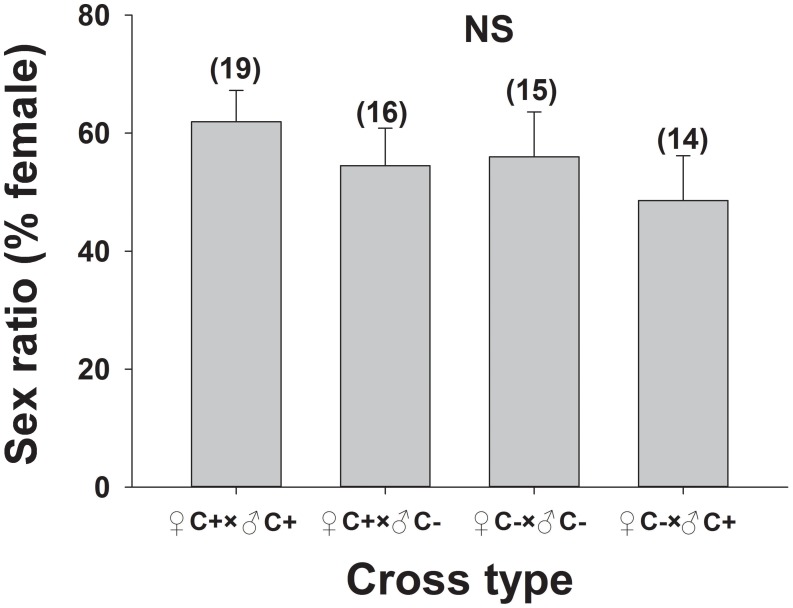
Sex ratio (proportion of females) in F1 offspring produced by crosses between *Cardinium*-infected (*C^+^*) and uninfected (*C^−^*) *B.tabaci* Q. Results are mean percent sex ratio ± SE. Number of each of the cross types are shown in parentheses. NS represent not significant at the 5% level (LSD test, *P*>0.05).

No significant differences were observed in the sex ratio of the F1 generation among the four possible crosses between *C* ^+^ and *C* ^−^ populations (*P*>0.05, ANOVA) (Fig. 2). These results suggest that no reproductive isolation or reproductive abnormalities occurred between *C* ^+^ and *C* ^−^ populations.

### *C* ^+^ and *C* ^−^ Population Parameters based on the Two-sex Life Table Method

**Figure 3:**
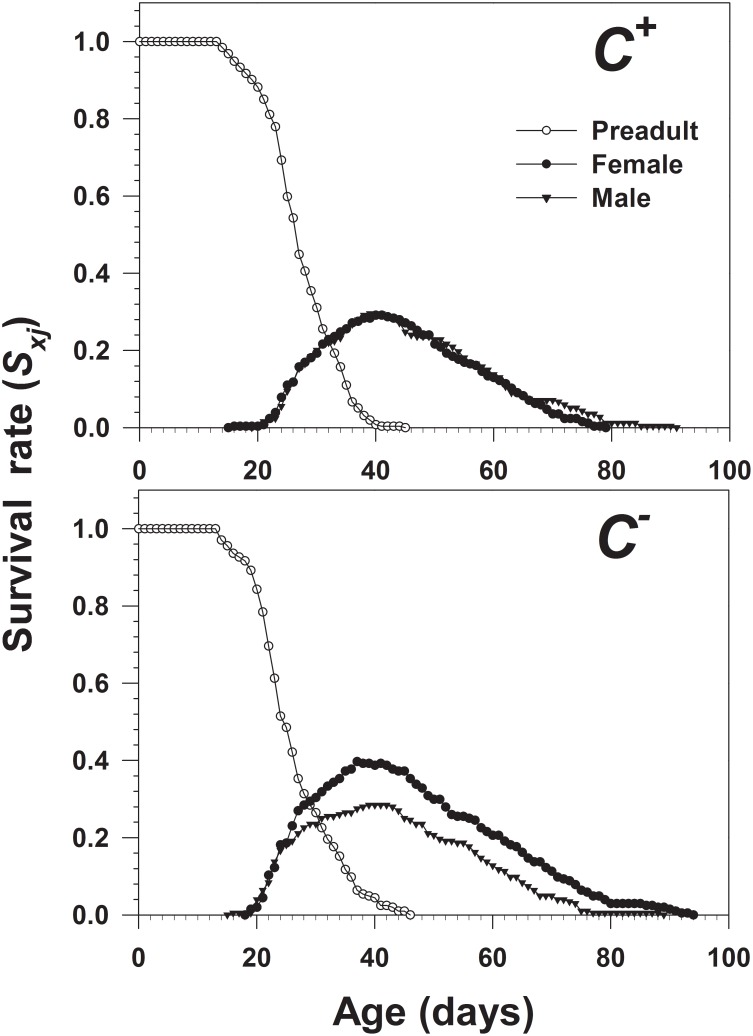
Age-stage specific survival rate (*s_xj_*) of *Cardinium*-infected (*C^+^*) and uninfected (*C^−^*) *B*. *tabaci* Q.

**Figure 4:**
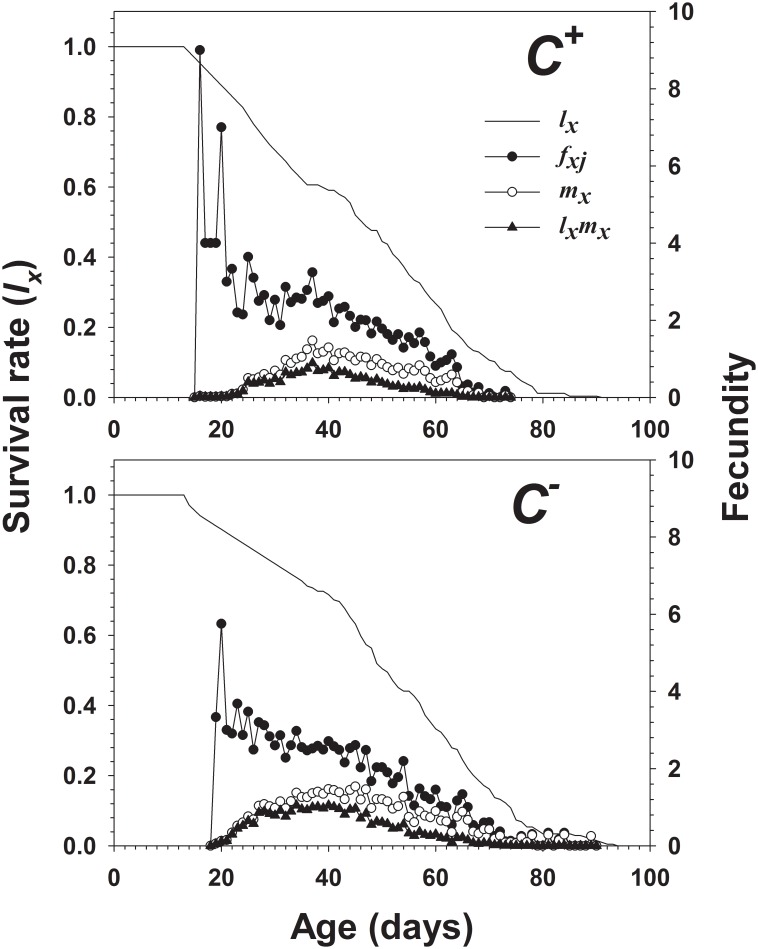
Age-specific survival rate (*l_x_*), female age-specific fecundity (*f_xj_*), age-specific fecundity of the total population (*m_x_*), and age-specific maternity (*l_x_m_x_*) of *Cardinium*-infected (*C^+^*) and uninfected (*C^−^*) *B*. *tabaci* Q.

The age-stage survival rate (*s_xj_*) showed the probability that a newly deposited egg of *B*. *tabaci* Q would survive to age *x* and stage *j* (Fig. 3). The mean number of offspring produced by a female adult at age *x* relative to the age-stage specific fecundity (*f_xj_*) is shown in Fig. 4. The maximum lifelong fecundity was found to be 152 eggs per female for the *C* ^+^ population and 192 eggs per female for the *C* ^−^ population.

**Figure 5:**
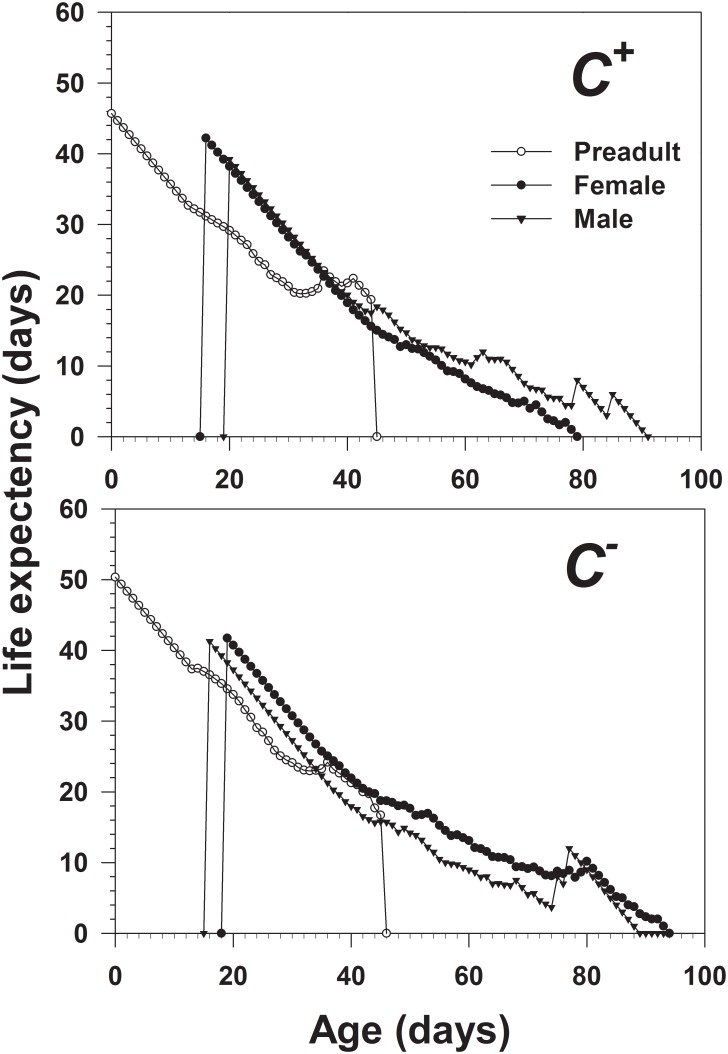
Age-stage specific life expectancies (*e_xj_*) of *Cardinium*-infected (*C^+^*) and uninfected (*C^−^*) *B*. *tabaci* Q.

The age-specific survival rate (*l_x_*) curve is the age-specific survival rate, including all individuals of the cohort and ignoring the stage of differentiation, while the age-specific fecundity (*m_x_*) is the mean fecundity of all individuals in the total population. The product of *l_x_* and *m_x_* is the age-specific maternity (*l_x_ m_x_*) of the *C* ^+^ and *C* ^−^ populations. Higher peaks of *m_x_* and *l_x_m_x_* were observed in the *C* ^−^ population. The age-stage specific life expectancy (*e_xj_*) of *B*. *tabaci* is shown in Fig. 5. The maximum life expectancy was 45.75 days for the *C* ^+^ population and 50.48 days for the *C* ^−^ population. The age-stage life expectancy of the *C* ^+^ population was significantly shorter than the *C* ^−^ population.

**Figure 6:**
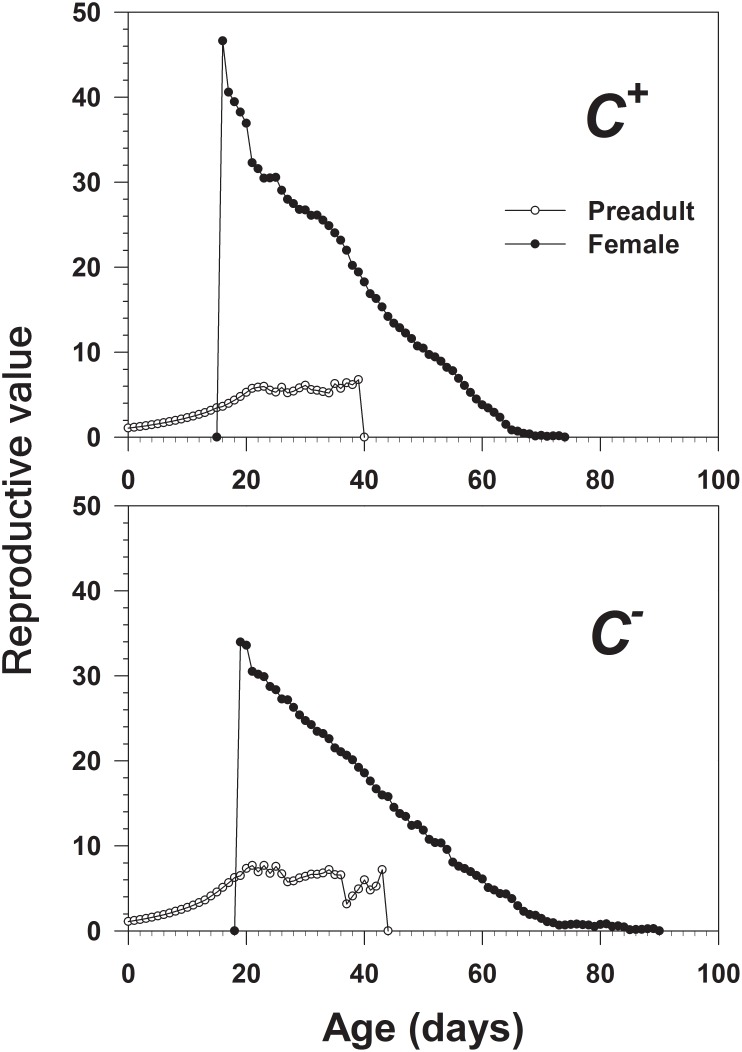
Age-stage specific reproductive value (*v_xj_*) of *Cardinium*-infected (*C^+^*) and uninfected (*C^−^*) *B*. *tabaci* Q.

The age-stage reproductive value (*v_xj_*) (Fig. 6) delineates the contribution of an individual to age *x* and stage *j* of the future population [40]. In our study, the reproductive values increased sharply to 47.23 in the *C* ^+^ population when females started to emerge at day 19. The corresponding reproductive values increased sharply to 33.96 in the *C* ^−^ population when females started to emerge at day 16, 3 days earlier than the *C* ^+^ population.

The fecundity and mean developmental time of each life stage, including pre-adult duration, adult (female, male) longevity, APOP (adult preoviposition period), TPOP (total preoviposition period), and oviposition period, are given in Table 1. The pre-adult duration of the *C* ^−^ population was significantly shorter than that of the *C* ^+^ population (*P*<0.05). However, differences in the other parameters between the two populations were not significant (*P*>0.05).

**Table 1:** Basic statistics of the life history of *Cardinium*-infected and uninfected *Bemisia tabaci* Q.

Statistic	Stage or sex	*C^+^*			*C^−^*		*P*
		n	Mean ± SE		n	Mean ± SE	
Pre-adult duration (d)	Pre-adult	155	28.97±0.43*		152	26.30±0.53	0.017
Adult longevity (d)	Female	75	29.79±1.06		87	33.37±1.51	0.05
	Male	80	29.94±1.24		65	30.37±1.29	0.81
TPOP (d)	Female	75	29.23±0.65		87	28.06±0.69	0.23
APOP (d)	Female	75	0.68±0.10		87	0.56±0.10	0.44
Oviposition (d)	Female	75	18.25±0.85		87	20.19±0.98	0.18
Fecundity (eggs per female)	Female	75	60.36±3.37		87	68.12±4.43	0.15

All *P* values are calculated using Student’s *t*-test. APOP (adult preoviposition period) and TPOP (total preoviposition period) were calculated using females that produced fertile eggs.

*Significant difference (Student’s *t*-test, *P*<0.05).

Based on the two-sex life table method (Table 2), the intrinsic rate of increase (*r*), net reproductive rate (*R*_0_), and finite rate of increase (*λ*) of the *C* ^−^ population (0.09192 d^−1^, 29.06 offspring, and 1.096 d^−1^, respectively) were significantly higher than that of the *C* ^+^ population (0.07561 d^−1^, 17.82 offspring, and 1.078 d^−1^, respectively). The mean generation time (*T*) of the *C* ^+^ population (38.09 d) was significantly longer than that of *C* ^−^ population (36.67 d).

**Table 2:** Population parameters for *Cardinium*-infected and uninfected *Bemisia tabaci* Q.

Population parameters	*C^+^* (SE)	*C^−^* (SE)
r (d−1)	0.07561 (0.0035)	0.09192 (0.0036)*
λ	1.078 (0.0037)	1.096 (0.012)*
R0	17.82 (2.04)	29.06 (3.04)*
T (d)	38.09 (0.82)*	36.67 (0.81)

*Significant difference (Student’s *t*-test, *P*<0.05)

## Discussion

Our results showed that the percentage of *C* ^−^ whitefly decreased after three generations, indicating that the *C* ^+^ whitefly has weak competitive ability. The results proved the initial hypothesis that the competitive ability of the infected host whiteflies was weaker than that of uninfected ones, which is in agreement with results of previous field surveys in China [8], [9]. Our results also revealed that no reproductive isolation occurred between the two populations and the *C* ^−^ population had higher fitnesses than the *C* ^+^ population. The *C* ^−^ whiteflies had higher *r* values (because they developed faster), higher survivorship of immature stages, and a higher net reproductive rate than those of *C* ^+^ whiteflies at 27°C. These differences may explain why *C* ^−^ whitefly populations have been dominant during the past few years in Shandong province, China.

For whiteflies, they possess a haplo-diploid sex determination system, in which unfertilized eggs developed into males and fertilized eggs develop into females [41]. In our study, we did not observe parthenogenesis or feminization in the experimental crosses. Although an analysis of *cytochrome P450* genes in the two whitefly populations revealed that they were highly similar, the effect of *Cardinium* on whitefly reproduction should be examined in more detail in future studies. In recent decades, multiple roles of bacterial symbionts in arthropods have been revealed [42], [43]. Bacterial symbionts can manipulate the reproductive biology of hosts or affect the host fitness to increase transmission of the symbiont [18], [21], [43]–[54]. Most studies have shown that *Cardinium* can alter the reproduction of its hosts, which, in turn, is helpful to the spread of the symbiont within the population [12]–[20], [41]. For instance, embryonic mortality resulting from cytoplasmic incompatibility is the most common effect associated with endosymbiont infection [50]; consequently, the symbionts can maximize their spread. Cytoplasmic incompatibility induced by *Cardinium* has been widely reported in arthropods, such as the parasitoid wasp *Encarsia pergandiella* [15], spider mite *Eotetranychus suginamensis* [16], sexual spider mite *Bryobia sarothamni* [55], and whitebacked planthopper *Sogatella furcifera* [17]. If *Cardinium* does not alter the reproduction of the whitefly host, then this symbiont may play a different role in the whitefly than observed in previous studies that showed it induces feminization [12], [13], parthenogenesis [14], and cytoplasmic incompatibility of infected hosts [15]–[17]. Our results are similar to the observations of White *et al*. [56], [57] and Stefanini & Duron [58], which showed there was no cytoplasmic incompatibility or progeny sex ratio distortion in *Cardinium*-infected individuals of the parasitic wasp *Encarsia inaron* [56], [57] or the spider *Holocnemus pluchei* [58].

Based on the two-sex life table method in this study, we revealed that the *C* ^−^ population had higher fitness than the *C* ^+^ population (Tables 1 and 2), which might indicate that *Cardinium* may have cryptic negative effects on the fitness of the host. The role *Cardinium* plays in changing the fitness cost of the whitefly should be examined further in future studies. Many studies have suggested that *Cardinium* can increase the fitness of the hosts [13], [18]–[20]. For example, Weeks & Stouthamer [18] found that the fecundity advantage of infected females was approximately 1.6 fold higher than that of uninfected females over a 6-day egg-laying period in the predatory mite, *Metaseiulus occidentailis*, and Giorgini *et al*. [13] showed that the antibiotic removal of *Cardinium* reduced offspring production by adult *Encarsia hispida* females. If it is the case that *Cardinium* has cryptic negative effects on the fitness of the whitefly host, then this would be in disagreement with previous studies that showed that *Cardinium* has no detectable effect on either reproduction or development of the host [56]–[59]. Similar negative effects of symbiont on host insect have been revealed for *Wolbachia* in *Drosophila*, in which the infection drastically reduces life span [60], and causes widespread degeneration of tissues, culminating in early death of *Drosophila melanogaster* [61]. On the other hand, because *Cardinium* still exists in the field, there might be a benefit to the whitefly. The evolution of this symbiont strain needs to be further explored.

Finally, the two-sex life table gives the most comprehensive description and analysis of the survival and reproduction of a population and, thus, this method may be highly beneficial in revealing the difference in the two populations. This method takes into account the male population and the variable developmental rate occurring among individuals and can overcome the shortcoming of the traditional female-based, age-specific life table method, which ignores the male individuals, the stage of differentiation, and variable developmental rates among individuals.

## Supporting Information

Table S1 The *cytochrome P450* genes identified in this study.
(XLS)

## References

[1] Oliveira M, Henneberry T, Anderson P (2001) History, current status, and collaborative research projects for *Bemisia tabaci* . Crop Prot 20: 709–723.

[2] Czosnek H, Ghanim M (2011) *Bemisia tabaci*-*Tomato yellow leaf curl virus* interaction causing worldwide epidemics. In: Winston M.O. Thompson. The whitefly, *Bemisia tabaci* (Homoptera: Aleyrodidae) interaction with geminivirus-infected host plants. Springer 51–67.

[3] De Barro PJ, Liu SS, Boykin LM, Dinsdale AB (2011) *Bemisia tabaci*: a statement of species status. Annu Rev Entomol 56: 1–19. 20690829 10.1146/annurev-ento-112408-085504

[4] Wang HL, Yang J, Boykin LM, Zhao QY, Li Q, et al (2013) The characteristics and expression profiles of the mitochondrial genome for the Mediterranean species of the *Bemisia tabaci* complex. BMC Genomics 14: 401. 23768425 10.1186/1471-2164-14-401 PMC3691742

[5] Chu D, Zhang YJ, Brown JK, Cong B, Xu BY, et al (2006) The introduction of the exotic Q biotype of *Bemisia tabaci* from the Mediterranean region into China on ornamental crops. Fla Entomol 89: 168–174.

[6] Chu D, Wan FH, Zhang YJ, Brown JK (2010) Change in the biotype composition of *Bemisia tabaci* in Shandong Province of China from 2005 to 2008. Environ Entomol 39: 1028–1036. 20550819 10.1603/EN09161

[7] Teng X, Wan FH, Chu D (2010) *Bemisia tabaci* biotype Q dominates other biotypes across China. Fla Entomol 93: 363–368.

[8] Pan HP, Chu D, Ge DQ, Wang SL, Wu QJ, et al (2011) Further spread of and domination by *Bemisia tabaci* biotype Q on field crops in China. J Econ Entomol 104: 978–985. 21735919 10.1603/ec11009

[9] Chu D, Gao CS, De Barro P, Zhang YJ, Wan FH (2011) Investigation of the genetic diversity of an invasive whitefly in China using both mitochondrial and nuclear DNA markers. Bull Entomol Res 101: 467–475. 21320364 10.1017/S0007485311000022

[10] Kurtti TJ, Munderloh UG, Andreadis TG, Magnarelli LA, Mather TN (1996) Tick cell culture isolation of an intracellular prokaryote from the tick *Ixodes scapularis.* . J Invertebrate Pathol 67: 318–321. 10.1006/jipa.1996.0050 8812616

[11] Zchori-Fein E, Perlman SJ, Kelly SE, Katzir N, Hunter MS (2004) Characterization of a ‘*Bacteroidetes*’ symbiont in *Encarsia* wasps (Hymenoptera: Aphelinidae): proposal of ‘Candidatus *Cardinium hertigii*’. Int J Syst Evol Microbiol 54: 961–968. 15143050 10.1099/ijs.0.02957-0

[12] Weeks AR, Marec F, Breeuwer JA (2001) A mite species that consists entirely of haploid females. Science 292: 2479–2482. 11431565 10.1126/science.1060411

[13] Giorgini M, Monti MM, Caprio E, Stouthamer R, Hunter MS (2009) Feminization and the collapse of haplodiploidy in an asexual parasitoid wasp harboring the bacterial symbiont *Cardinium* . Heredity 102: 365–371. 19190669 10.1038/hdy.2008.135

[14] Provencher LM, Morse GE, Weeks AR, Normark BB (2005) Parthenogenesis in the *Aspidiotus nerii* complex (Hemiptera: Diaspididae): a single origin of a worldwide, polyphagous lineage associated with *Cardinium* bacteria. Ann Entomol Soc Amer 98: 629–635.

[15] Hunter MS, Perlman SJ, Kelly SE (2003) A bacterial symbiont in the Bacteroidetes induces cytoplasmic incompatibility in the parasitoid wasp *Encarsia pergandiella* . Proc R Soc Lond Ser B-Biol Sci 270: 2185–2190. 10.1098/rspb.2003.2475 PMC1691482 14561283

[16] Gotoh T, Noda H, Ito S (2007) *Cardinium* symbionts cause cytoplasmic incompatibility in spider mites. Heredity 98: 13–20. 17035954 10.1038/sj.hdy.6800881

[17] Zhang XF, Zhao DX, Hong XY (2012) *Cardinium*-the leading factor of cytoplasmic incompatibility in the planthopper *Sogatella furcifera* doubly infected with *Wolbachia* and *Cardinium* . Environ Entomol 41: 833–840.

[18] Weeks AR, Stouthamer R (2004) Increased fecundity associated with infection by a cytophaga-like intracellular bacterium in the predatory mite, *Metaseiulus occidentalis* . Proc R Soc Lond Ser B 271: S193. 10.1098/rsbl.2003.0137 PMC1810030 15252981

[19] Zchori-Fein E, Gottlieb Y, Kelly S, Brown J, Wilson J, et al (2001) A newly discovered bacterium associated with parthenogenesis and a change in host selection behavior in parasitoid wasps. Proc Natl Acad Sci USA 98: 12555–12560. 11592990 10.1073/pnas.221467498 PMC60092

[20] Kenyon S, Hunter M (2007) Manipulation of oviposition choice of the parasitoid wasp, *Encarsia pergandiella*, by the endosymbiotic bacterium *Cardinium* . J Evol Biol 20: 707–716. 17305836 10.1111/j.1420-9101.2006.01238.x

[21] Harris L, Kelly S, Hunter M, Perlman S (2010) Population dynamics and rapid spread of *Cardinium*, a bacterial endosymbiont causing cytoplasmic incompatibility in *Encarsia pergandiella* (Hymenoptera: Aphelinidae). Heredity 104: 239–246. 19812617 10.1038/hdy.2009.130

[22] Chu D, Gao C, De Barro P, Zhang Y, Wan F, et al (2011) Further insights into the strange role of bacterial endosymbionts in whitefly, *Bemisia tabaci*: Comparison of secondary symbionts from biotypes B and Q in China. Bull Entomol Res 101: 477–486. 21329550 10.1017/S0007485311000083

[23] Pan H, Li X, Ge D, Wang S, Wu Q, et al (2012) Factors affecting population dynamics of maternally transmitted endosymbionts in *Bemisia tabaci* . PLoS ONE 7: e30760. 22383972 10.1371/journal.pone.0030760 PMC3285672

[24] Chi H (1988) Life-table analysis incorporating both sexes and variable development rates among individuals. Environ Entomol 17: 26–34.

[25] Nirgianaki A, Banks GK, Frohlich DR, Veneti Z, Braig HR, et al (2003) *Wolbachia* infections of the whitefly *Bemisia tabaci.* . Curr Microbiol 47: 93–101. 14506854 10.1007/s00284-002-3969-1

[26] Zchori-Fein E, Brown J (2002) Diversity of prokaryotes associated with *Bemisia tabaci* (Gennadius) (Hemiptera: Aleyrodidae). Ann Entomol Soc Am 95: 711–718.

[27] Chiel E, Gottlieb Y, Zchori-Fein E, Mozes-Daube N, Katzir N, et al (2007) Biotype-dependent secondary symbiont communities in sympatric populations of *Bemisia tabaci* . Bull Entomol Res 97: 407–413. 17645822 10.1017/S0007485307005159

[28] Simon C, Frati F, Beckenbach A, Crespi B, Liu H, et al (1994) Evolution, weighting, and phylogenetic utility of mitochondrial gene sequences and a compilation of conserved polymerase chain reaction primers. Ann Entomol Soc Am 87: 651–701.

[29] Chu D, Hu XS, Gao CS, Zhao HY, Nichols RL, et al (2012) Use of mtCOI PCR-RFLP for identifying subclades of *Bemisia tabaci* Mediterranean group. J Econ Entomol 105: 242–251. 22420277 10.1603/ec11039

[30] Khasdan V, Levin I, Rosner A, Morin S, Kontsedalov S, et al (2005) DNA markers for identifying biotypes B and Q of *Bemisia tabaci* (Hemiptera: Aleyrodidae) and studying population dynamics. Bull Entomol Res 95: 605–613. 16336708 10.1079/ber2005390

[31] Nakamura Y, Kawai S, Yukuhiro F, Ito S, Gotoh T, et al (2009) Prevalence of *Cardinium* bacteria in planthoppers and spider mites and taxonomic revision of "*Candidatus* Cardinium hertigii" based on detection of a new *Cardinium* group from biting midges. Appl Environ Microbiol 75: 6757–6763. 19734338 10.1128/AEM.01583-09 PMC2772453

[32] Grabherr MG, Haas BJ, Yassour M, Levin JZ, Thompson DA, et al (2011) Full-length transcriptome assembly from RNA-Seq data without a reference genome. Nat Biotechnol 29: 644–652. 21572440 10.1038/nbt.1883 PMC3571712

[33] Wang XW, Luan JB, Li JM, Su YL, Xia J, et al (2011) Transcriptome analysis and comparison reveal divergence between two invasive whitefly cryptic species. BMC Genomics 12: 458. 21939539 10.1186/1471-2164-12-458 PMC3189941

[34] Luan JB, Ruan YM, Zhang L, Liu SS (2008) Pre-copulation intervals, copulation frequencies, and initial progeny sex ratios in two biotypes of whitefly, *Bemisia tabaci.* . Entomol Exp Appl 129: 316–324.

[35] Li X, Degain BA, Harpold VS, Marçon PG, Nichols RL, et al (2012) Baseline susceptibilities of B-and Q-biotype *Bemisia tabaci* to anthranilic diamides in Arizona. Pest Manag Sci 68: 83–91. 21714059 10.1002/ps.2227

[36] Chi H (2013) TWOSEX-MsChart: a computer program for the age-stage, two-sex life table analysis. http://140.120.197.173/Ecology/download/Twosex-MSChart.rar.

[37] Chi H, Su HY (2006) Age-stage, two-sex life tables of *Aphidius gifuensis* (Ashmead) (Hymenoptera: Braconidae) and its host *Myzus persicae* (Sulzer) (Homoptera: Aphididae) with mathematical proof of the relationship between female fecundity and the net reproductive rate. Environ Entomol 35: 10–21.

[38] Efron B, Tibshirani R (1993) An introduction to the bootstrap. London: Chapman & hall.

[39] Goodman D (1982) Optimal life histories, optimal notation, and the value of reproductive value. Am Nat 803–823.

[40] Fisher RA (1930) The genetical theory of natural selection. Clarendon Press, Oxford, United Kingdom.

[41] Blackman RL, Cahill M (1998) The karyotype of *Bemisia tabaci* (Hemiptera: Aleyrodidae). Bull Entomol Res 88: 213–215.

[42] Werren JH (1997) Biology of *Wolbachia* . Annu Rev Entomol 42: 587–609. 15012323 10.1146/annurev.ento.42.1.587

[43] Werren JH, Baldo L, Clark ME (2008) *Wolbachia*: master manipulators of invertebrate biology. Nat Rev Microbiol 6: 741–751. 18794912 10.1038/nrmicro1969

[44] Hagimori T, Abe Y, Date S, Miura K (2006) The first finding of a *Rickettsia* bacterium associated with parthenogenesis induction among insects. Curr Microbiol 52: 97–101. 16450063 10.1007/s00284-005-0092-0

[45] Haine ER (2008) Symbiont-mediated protection. Proc R Soc B 275: 353–361. 10.1098/rspb.2007.1211 PMC2213712 18055391

[46] Wang JJ, Dong P, Xiao LS, Dou W (2008) Effects of removal of *Cardinium* infection on fitness of the stored-product pest *Liposcelis bostrychophila* (Psocoptera: Liposcelididae). J Econ Entomol 101: 1711–1717. 18950056 10.1603/0022-0493(2008)101[1711:eoroci]2.0.co;2

[47] Jaenike J, Unckless R, Cockburn SN, Boelio LM, Perlman SJ (2010) Adaptation via symbiosis: recent spread of a *Drosophila* defensive symbiont. Science 329: 212–215. 20616278 10.1126/science.1188235

[48] Himler AG, Adachi-Hagimori T, Bergen JE, Kozuch A, Kelly SE, et al (2011) Rapid spread of a bacterial symbiont in an invasive whitefly is driven by fitness benefits and female bias. Science 332: 254–256. 21474763 10.1126/science.1199410

[49] Vanthournout B, Swaegers J, Hendrickx F (2011) Spiders do not escape reproductive manipulations by *Wolbachia* . BMC Evol Biol 11: 15. 21235755 10.1186/1471-2148-11-15 PMC3025852

[50] Rousset F, Bouchon D, Pintureau B, Juchault P, Solignac M (1992) *Wolbachia* endosymbionts responsible for various alterations of sexuality in arthropods. Proc R Soc Lond Ser B-Biol Sci 250: 91–98. 10.1098/rspb.1992.0135 1361987

[51] Stouthamer R, Breeuwer JA, Hurst GD (1999) *Wolbachia pipientis*: microbial manipulator of arthropod reproduction. Annu Rev Microbiol 53: 71–102. 10547686 10.1146/annurev.micro.53.1.71

[52] Stouthamer R, Breeuwer J, Luck R, Werren J (1993) Molecular identification of microorganisms associated with parthenogenesis. Nature 361: 66–68. 7538198 10.1038/361066a0

[53] Hurst GD, Jiggins FM, von der Schulenburg JHG, Bertrand D, West SA, et al (1999) Male-killing *Wolbachia* in two species of insect. Proc R Soc Lond Ser B 266: 735–740.

[54] Breeuwer J, Stouthamer R, Barns S (1992) Phylogeny of cytoplasmic incompatibility micro-organisms in the parasitoid wasp genus *Nasonia* (Hymenoptera: Pteromalidae) based on 16S ribosomal DNA sequences. Insect Mol Biol 1: 25–36. 1343772 10.1111/j.1365-2583.1993.tb00074.x

[55] Ros VID, Breeuwer JAJ (2009) The effects of, and interactions between, *Cardinium* and *Wolbachia* in the doubly infected spider mite *Bryobia sarothamni* . Heredity 102: 413–422. 19223923 10.1038/hdy.2009.4

[56] White JA, Kelly SE, Perlman SJ, Hunter MS (2009) Cytoplasmic incompatibility in the parasitic wasp *Encarsia inaron*: disentangling the roles of *Cardinium* and *Wolbachia* symbionts. Heredity 102: 483–489. 19223921 10.1038/hdy.2009.5 PMC4150353

[57] White JA, Kelly SE, Cockburn SN, Perlman SJ, Hunter MS (2011) Endosymbiont costs and benefits in a parasitoid infected with both *Wolbachia* and *Cardinium.* . Heredity 106: 585–591. 20606691 10.1038/hdy.2010.89 PMC3183907

[58] Stefanini A, Duron O (2012) Exploring the effect of the *Cardinium* endosymbiont on spiders. J Evol Biol 25: 1521–1530. 22591396 10.1111/j.1420-9101.2012.02535.x

[59] Bull JJ (1983) Evolution of sex determining mechanisms. Menlo Park (California): Benjamin/Cummings.

[60] Brownstein J, Hett E, O’Neill S (2003) The potential of virulent *Wolbachia* to modulate disease transmission by insects. J Invertebrate Pathol 84: 24–29. 10.1016/s0022-2011(03)00082-x 13678709

[61] Min KT, Benzer S (1997) Genetics *Wolbachia*, normally a symbiont of *Drosophila*, can be virulent, causing degeneration and early death. Proc Natl Acad Sci 94: 10792–107961. 9380712 10.1073/pnas.94.20.10792 PMC23488

